# Pulmonary Tuberculosis Versus Recurrent Chemotherapy-Induced Pneumonitis: A Clinical Dilemma

**DOI:** 10.7759/cureus.1742

**Published:** 2017-10-04

**Authors:** Gulrayz Ahmed, Muhammad W Saif

**Affiliations:** 1 Medicine, Mount Auburn Hospital; 2 Hematology/Oncology, Tufts Medical Center

**Keywords:** pancreatic cancer, irinotecan, tuberculosis, latent tuberculosis, pneumonitis, chemotherapy, gemcitabine, oxaliplatin

## Abstract

Chemotherapy-induced lung toxicity can affect pulmonary parenchyma, pleura, airways, pulmonary vascular system, mediastinum or the neuromuscular system that is responsible for respiration. Chemotherapy-induced pulmonary toxicity is a diagnosis of exclusion. When the patients with malignancies develop pulmonary toxicity such as pneumonitis and distinguishing it from alternative diseases such as infectious, thrombotic, cardiac, malignant or exacerbation of chronic lung conditions can be difficult. Moreover, such patients are often immunosuppressed, physically stressed from the underlying disease and the cancer treatment and hence, more susceptible to usual and unusual or opportunistic infections. We describe a patient with pancreatic cancer who was assumed to develop recurrent chemotherapy-induced pneumonitis to various agents, including irinotecan and docetaxel, but subsequently proved to have reactivation of tuberculosis (TB). With tuberculosis not being uncommon in cancer patients, we now believe that his symptoms could all have been because of an active tuberculosis infection, especially with his latent TB history and pulmonary symptoms. Information about the link between the treatment of solid-organ cancers and TB is very limited. Our case underlines the recognition about this link of chemotherapy and TB as well as remind us of the lack of widely accepted and established standards for both screenings for latent TB and for the treatment of active TB in the patients undergoing systemic treatment. A simple test such as a tuberculin skin test or QuantiFERON-TB Gold test can be used to rule out latent TB before beginning radiotherapy or chemotherapy in these patients. Clinicians must be cognizant of this condition to prevent further morbidity and mortality in these cancer patients and include activated TB in the differential diagnosis of pulmonary toxicity suspected in a patient undergoing chemotherapy with unexplained pulmonary findings.

## Introduction

Chemotherapy-induced pneumonitis, a non-infectious inflammation of the lungs is a known toxicity associated with several anti-cancer drugs, including bleomycin, busulfex (busulfan), cyclophosphamide, bis-chloroethylnitrosourea BiCNU (carmustine), taxanes, Methotrexate, gemcitabine, mTOR inhibitors, epidermal growth factor receptor (EGFR) inhibitors, CTLA4 or anti-Programmed Cell Death Protein (PD-1/PD-L1) inhibitors, as well as radiation therapy [1-3]. Taghian, et al. reported that 14.6% of the patients receive the concomitant chemotherapy and radiation therapy for breast cancer developed pneumonitis [4]. Irinotecan, a topoisomerase poison has also been associated with lung injury, including pneumonitis and fibrosis [5-7]. The hallmark manifestations of pneumonitis include shortness of breath, dry cough, low-grade fever, chest tightness, chest pain and general malaise [1-7]. They range from mild to severe respiratory symptoms but may not appear until weeks to months into treatment and sometimes long after the end of treatments, leading to frequent misdiagnoses particularly because the symptoms resemble those of pneumonia. Pneumonitis is a clinical diagnosis and no specific diagnostic test is available. The computed tomography (CT) scan may show diffuse ground-glass changes accompanied by thickened septal lines, interstitial infiltrates, or diffuse alveolar infiltrates [2-3]. The treatment is aimed at reducing inflammation by using steroids such as prednisone (often taken for up to 10 weeks with the dosage tapered over time) [1]. Though the majority of the patients achieve complete resolution for their symptoms, they are still prone to develop subsequent pulmonary complications, including fibrosis, a permanent scarring of the lungs. However, certain agents such as bleomycin are associated with permanent effects and new targeted agents such as Epidermal Growth Factor Receptor (EGFR) or anti-Programmed Cell Death Protein (PD-1) inhibitors have resulted in mortality [1-3,5].

But on the other hand, it is an established fact that certain malignancies can predispose patients to have active or reactivation of tuberculosis (TB), at the time of diagnosis or during the treatment [8-10]. In such patients, the diagnosis can even be more cumbersome, especially the TB is not that commonly seen in the United States and can go unnoticed. We present a grey zone case that was suspected to have recurrent chemotherapy-induced pneumonitis with known latent TB infection (LTBI) but later found to have active TB that was improving with steroid pulse doses (steroids have shown to reduce inflammatory processes caused by Mycobacterium in multiple studies) and recurring with different agents administered to the patient [11].

## Case presentation

A 64-year-old Asian male with a significant past medical history of Graves disease, iron deficiency, anemia, and diabetes mellitus, presented to the primary care physician (PCP) with complaints of weight loss, yellow discoloration of the skin, foul smelling and fatty stools for a few weeks. He was a former smoker, with a smoking history of 25 years and stopped almost the year before. His family history was significant for colon cancer in his mother and uncle, with both dying in their late 70s.

During the physical examination, he appeared jaundiced but otherwise unremarkable for any abdominal tenderness or mass or palpation. Basic laboratory workup including complete blood count, basic metabolic panel, liver function tests and lipase were ordered, which came up abnormal for mild anemia (hemoglobin: 9.1 g/dL) and obstructive jaundice pattern with aspartate aminotranferase (AST) (IU/L): 52, alanine aminotransferase (ALT) (IU/L): 87 H, lactate dehydrogenase (IU/L): 167, alkaline phosphatase (ALK) (IU/L): 226 H, and total bilirubin (MG/DL): 0.7. To alleviate his symptoms, his PCP started him on pancreatic enzyme replacement after which the patient reported some improvement in his fatty stool production. With concerns about his weight loss and obstructive jaundice, the computed tomography (CT) scan of the abdomen and pelvis was done which showed multiple pancreatic duct stones, with the largest measuring around 2.2 cm in the region of ampulla, with common bile duct and pancreatic duct obstruction and diffuse atrophy of the pancreatic parenchyma, but no discrete lesions. He underwent therapeutic Endoscopic retrograde cholangiopancreatography (ERCP) to remove the stones but it was unsuccessful, requiring him to have a percutaneous biliary stent placed to help with his obstructive jaundice.

The CT scan of the chest showed multiple nodular opacities on the lung bases for which he was referred to the pulmonology department. An extensive workup was performed to rule out infectious versus autoimmune inflammatory process or malignancy based on his history of smoking. He underwent a bronchoscopy and bronchoalveolar lavage (BAL), which was negative. Fine Needle Aspiration (FNA) of the right lower lobe lesion was positive for malignancy, revealing well-differentiated adenocarcinoma with mucin cytokeratin (CK-7) positive, thyroid transcription factor 1 (TTF- 1) positive, Napsin A positive, homeobox protein (CDX-2) positive with CK-20 and cancer antigen (CA) 19-9 negative. It supported the diagnosis of malignancy, consistent with his primary tumor in the pancreas. He underwent a positron emission tomography-computed tomography (PET/CT) revealing pleural-based mass at the left lateral lung base with standardized uptake values (SUV) of 7.15 and increased uptake in the small bowel in the middle-to-upper abdomen with SUV 9.43. His cancer antigen 19-9 work up was negative while his cancer antigen 125 (CA 125) levels were elevated (80 U/mL) with increased carcinoembryonic antigen (CEA) (17.5 ng/mL). After a multidisciplinary discussion, it was concluded that the patient has stage IV pancreatic cancer with lung metastases.

**Figure 1:**
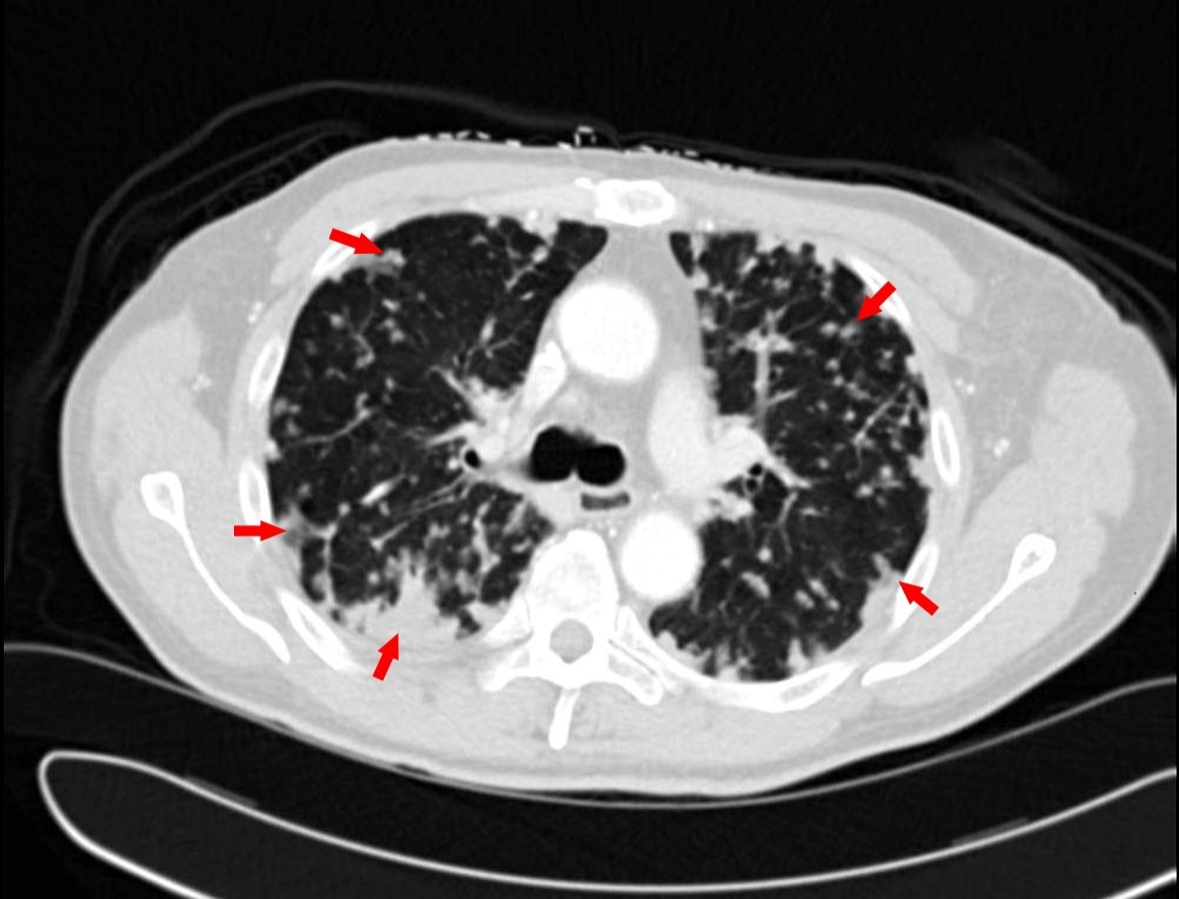
Computed tomography of the chest showing scattered interstitial opacities suggestive of pneumonitis, some labeled with arrows.

He was initially treated with Gemcitabine, Oxaliplatin, 5-Fluorouracil and Flonoic Acid (GOLF) chemotherapy regimen for his stage IV pancreatic cancer. After 12 cycles (six months), he developed grade two fatigue along with grade three peripheral neuropathy and grade three thrombocytopenia. He was then changed to a regimen including folinic acid, 5-fluorouracil, and irinotecan (FOLFIRI) chemotherapy. After three cycles (six weeks), he reported the gradual onset of shortness of breath (SOB), associated with a dry cough and low-grade fevers. He presented to the PCP's office and was found to be tachycardic as well as hypoxic to mid-80s of oxygen saturation. He was immediately sent to the emergency department and underwent a chest computed tomography (CT) which showed interval change with scattered interstitial opacities with ground glass appearance suggestive of chemotherapy-induced pneumonitis (Figure 1). Otherwise, his cancer burden was stable when compared to the previous scans. A pulmonologist was also involved and they performed a repeat bronchoscopy, which failed to reveal any significant results including negative microbiology. He was started on steroids after which his symptoms drastically improved and was eventually discharged. Unfortunately, after recovering, he was re-admitted with almost identical symptoms within a week, but this time, he was found to have Haemophilus influenzae. He was discharged after improvement and was on home oxygen which he continued to use intermittently.

His oxygen requirement continued to increase, but he showed the willingness to continue treatment for his pancreatic malignancy, and with complications to GOLF and irinotecan from FOLFIRI, he was started on docetaxel monotherapy at low doses of only 25mg/m^2 ^weekly. Unfortunately, docetaxel had to be changed to every other week on cycle two at day eight due to persistent and worsening SOB. Two weeks later, his SOB worsened along with a productive cough and the status of the performance declined. He was again evaluated for underlying etiology. The review of his extensive workup showed a positive QuantiFERON gold test (QFT), the Food and Drug Administration (FDA) approved the test for tuberculosis infection or latent tuberculosis and elevated antinuclear antibody (ANA) titers of 1:80. Considering the productive cough, history of weight loss and positive QuantiFERON gold test, he seemed to have active TB. He underwent CT scan of the chest, which again showed innumerable nodules with peripheral predominance, more in the bases as compared to the apices. One out of three acid-fast bacilli (AFB) sputum smears were positive but species was unidentifiable. The infectious disease team was also involved and he was placed on isoniazid and pyridoxine. However, his overall performance status, fatigue, and pulmonary status continued to deteriorate. After the approval from his family, the patient was eventually placed on comfort measures.

## Discussion

Interstitial lung diseases are diffuse parenchymal lung diseases representing a heterogeneous group of disorders. Most of the interstitial disorders are associated with a restrictive pattern with the reduction in the total lung capacity, functional residual capacity, and residual volume. Some chemotherapeutic agents are also known to cause pneumonitis and other forms of lung toxicity [1-7]. Chemotherapy-induced pulmonary toxicity is a diagnosis of exclusion. The usual differential diagnosis includes infectious pneumonia, pulmonary embolus, cardiac-related respiratory distress, malignancy, lymphangitic carcinomatosis or exacerbation of chronic lung conditions and distinguishing them from interstitial pneumonitis can be challenging. However, our case brings an important message about the hidden diagnosis among the other problems from the clinical picture, the reactivation of TB.

Although it has been well recognized that hematologic malignancies increase the risk of developing active TB, the link between solid-organ cancers and developing TB is less known. Different case series of individual institutions have shown conflicting results with some showing an increase in the risk of developing active TB in solid cancer patients compared to those without cancer by 4.7, while the others ruling out any such association [8]. It was also noticed that most of these patients were either immigrant to the United States of America or were receiving higher doses of steroids [8,11].

This link is further complicated by the fact that the current US guidelines for the management of latent tuberculosis infection in cancer patients date back to the studies conducted in the 1970s but during the last four decades, the skyline of chemotherapy and biotherapy has changed tremendously.

We chose to publish our case for discussion as well as to bring an awareness to the physicians caring such patients. This was an interesting case not only because of the fact that he had an underlying LTBI but also was diagnosed with chemotherapy-induced pneumonitis (secondary to irinotecan, docetaxel) twice, as both the clinical and radiological findings were suggestive and he did respond to steroids both the times. With TB not being uncommon in cancer patients, we now believe that his symptoms could have been because of an active TB infection, especially with his LTBI history and pulmonary symptoms. Significant literature exists where reactivation of LTBI has been reported for the patients undergoing chemotherapy. Moreover, such patients are often immunosuppressed, physically stressed from the underlying disease along with the cancer treatment and hence more susceptible to opportunistic infections (particularly respiratory).

Many possible mechanisms have been suggested to explain the link between chemotherapy for solid tumors and TB which includes the local and systemic causes.

The local causes may include pulmonary toxicity secondary to chemotherapy (allergic reaction, oxidant injury, pulmonary vascular damage, deposition of phospholipids within cells, immune-mediated injury, or direct injury), radiation-induced lung injury (acute or chronic pulmonary toxicity injury in non-neoplastic type I and II pneumocytes, endothelial cells and stromal fibroblasts), underlying primary or metastatic malignancy.

The systemic causes may include immunosuppression by chemotherapy or malignancy, steroids, and other systemic factors.

Another unique aspect of this case is that the incidence of irinotecan -induced pneumonitis was previously reported only in the Asian population. Is there an ethnic predisposition?. This observation itself needs attention in the future studies. The patient was heterozygous for the TA7 polymorphism.

The Centers for Disease Control and Prevention and the American Thoracic Society recognize cancer as a risk factor for developing TB. Our case further suggests that LTBI reactivation can occur in immunosuppressed individuals and those who are receiving systemic chemotherapy with or without radiation.

## Conclusions

Information about the link between solid-organ cancers treatment and TB is very limited. Our patient had pancreatic carcinoma which spread to the lungs and was initially started on Gemcitabine, Oxaliplatin, 5-Fluorouracil and Flonoic Acid (GOLF) and transitioned to second-line chemotherapy fluorouracil and irinotecan (FOLFIRI) because of the side effects. He subsequently developed TB. Given the likelihood of underlying LTBI in this patient prior to the initiation of chemotherapy, it is postulated that his indolent/inactive TB converted to active TB possibly secondary to both local tissues, e.g lungs metastases, pneumonitis due to chemotherapy and systemic causes, e.g.: immunosuppression and steroids. Our case underlines the recognition about this link of chemotherapy and TB and TB should be included in the differential diagnosis of unexplained pulmonary findings in a patient with the history of LTBI.
